# Social Cognition and Behavioral Assessments Improve the Diagnosis of Behavioral Variant of Frontotemporal Dementia in Older Peruvians With Low Educational Levels

**DOI:** 10.3389/fneur.2021.704109

**Published:** 2021-09-06

**Authors:** Nilton Custodio, Rosa Montesinos, Lizardo Cruzado, Eder Herrera-Perez, Virgilio E. Failoc-Rojas, Maritza Pintado-Caipa, Wendy Seminario G, José Cuenca, Carlos Gamboa, Monica M. Diaz

**Affiliations:** ^1^Servicio de Neurología, Instituto Peruano de Neurociencias, Lince, Peru; ^2^Unidad de Diagnóstico de Deterioro Cognitivo y Prevención De Demencia, Instituto Peruano de Neurociencias, Lince, Peru; ^3^Unidad de Investigación, Instituto Peruano de Neurociencias, Lince, Peru; ^4^Facultad de Medicina, Universidad Peruana Cayetano Heredia, Lima, Peru; ^5^Instituto Nacional de Salud Mental “Honorio Delgado—Hideyo Noguchi”, Lima, Peru; ^6^Grupo de investigación Molident, Universidad San Ignacio de Loyola, Lima, Peru; ^7^Unidad de Investigación para la Generación y Síntesis de Evidencias en Salud, Universidad San Ignacio de Loyola, Lima, Peru; ^8^Atlantic Fellow, Global Brain Health Institute, University of California, San Francisco, San Francisco, CA, United States; ^9^Servicio de Neuropsicología, Instituto Peruano de Neurociencias, Lima, Peru; ^10^Carrera de Psicología, Facultad de Ciencias de la Salud, Universidad Privada del Norte, Lima, Peru; ^11^Department of Neurology, University of North Carolina at Chapel Hill, Chapel Hill, NC, United States; ^12^Facultad de Salud Pública y Administración, Universidad Peruana Cayetano Heredia, Lima, Peru

**Keywords:** social cognition, behavioral scales, frontotemporal dementia, low education, screening

## Abstract

**Background:** The behavioral variant of frontotemporal dementia (bvFTD), characterized by early behavioral abnormalities and late memory impairment, is a neurodegenerative disorder with a detrimental impact on patients and their caregivers. bvFTD is often difficult to distinguish from other neurodegenerative diseases, such as Alzheimer's disease (AD), using brief cognitive tests. Combining brief socio-cognitive and behavioral evaluations with standard cognitive testing could better discriminate bvFTD from AD patients. We sought to evaluate the diagnostic accuracy of brief socio-cognitive tests that may differentiate bvFTD and AD patients with low educational levels.

**Methods:** A prospective study was performed on 51 individuals over the age of 50 with low educational levels, with bvFTD or AD diagnosed using published criteria, and who were receiving neurological care at a multidisciplinary neurology clinic in Lima, Peru, between July 2017 and December 2020. All patients had a comprehensive neurological evaluation, including a full neurocognitive battery and brief tests of cognition (Addenbrooke's Cognitive Examination version III, ACE-III), social cognition (Mini-social Cognition and Emotional Assessment, Mini-SEA), and behavioral assessments (Frontal Behavioral Inventory, FBI; Interpersonal Reactivity Index—Emphatic Concern, IRI-EC; IRI—Perspective Taking, IRI-PT; and Self-Monitoring Scale—revised version, r-SMS). Receiver operating characteristic (ROC) analysis to calculate the area under the curve (AUC) was performed to compare the brief screening tests individually and combined to the gold standard of bvFTD and AD diagnoses.

**Results:** The AD group was significantly older than the bvFTD group (*p* < 0.001). An analysis of the discriminatory ability of the ACE-III to distinguish between patients with AD and bvFTD (AUC = 0.85) and the INECO Frontal Screening (IFS; AUC = 0.78) shows that the former has greater discriminatory ability. Social and behavioral cognition tasks were able to appropriately discriminate bvFTD from AD. The Mini-SEA had high sensitivity and high moderate specificity (83%) for discriminating bvFTD from AD, which increased when combined with the brief screening tests ACE-III and IFS. The FBI was ideal with high sensitivity (83%), as well as the IRI-EC and IRI-PT that also were adequate for distinguishing bvFTD from AD.

**Conclusions:** Our study supports the integration of socio-behavioral measures to the standard global cognitive and social cognition measures utilized for screening for bvFTD in a population with low levels of education.

## Introduction

The prevalence of frontotemporal dementia, a neurodegenerative disease characterized by difficulties with memory often preceded by significant behavioral changes, has been reported to range from two in 100,000 to 31 in 100,000 (1). Although a rare neurodegenerative disorder, it can have a detrimental impact on patients and their caregivers given the significant associated early behavioral abnormalities that can impede activities of daily living, decrease the quality of life of the patient, and increase caregiver burden (2, 3). Frontotemporal dementia is characterized by two distinct syndromes presenting with differing clinical symptoms and regional cerebral atrophy patterns on neuroimaging. The first syndrome, characterized by prominent abnormal behavioral symptoms, is called the behavioral variant of frontotemporal dementia (bvFTD). The second, primary progressive aphasia, is characterized by an abnormal language pattern but less so by behavioral disturbances (4). Patients with bvFTD are frequently misdiagnosed with a primary psychiatric disorder or a neurological syndrome with a frontal lobe syndrome leading to behavioral disturbances (5, 6). Given the extensive differential diagnosis for bvFTD, its rarity, and its detrimental impact on the quality of life, it is crucial to identify the disease early on in its course to offer appropriate counseling, monitoring, and prognostication to patients, families, and caregivers. More sensitive and specific screening tools are needed to correctly diagnose this disorder in the clinical setting and differentiate it from other dementias, such as Alzheimer's disease (AD) or primary psychiatric disorders.

The presenting symptoms in the early stages of bvFTD are behavioral and personality changes and executive function difficulties, with memory impairment occurring in more advanced stages of the disease (4). Apathy in bvFTD manifests as poor motivation, lack of interest in previously enjoyable activities, and progressive social isolation, which is often misdiagnosed as depression (7). Disinhibition may coexist with apathy that is often mistaken for mania or hypomania, obsessive–compulsive disorder, or a personality disorder (8). Disinhibition leads to impulsivity, manifesting as an inability to express oneself in a socially acceptable manner, excessive spending, inappropriate sexual acts, or socially embarrassing behaviors (i.e., childish behaviors, excessive and inappropriate familiarity with strangers, and disobedience of socially appropriate rules) (9). In some patients with bvFTD, the first symptoms are pathological gambling (10) or hyper-religiosity (11, 12). In other patients, the first symptoms may be stereotyped behaviors, including repetitive motor routines or more complex obsessions (13). Moreover, patients may have altered eating habits, such as increased appetite, ingesting food between meals, or overeating at meals that does not adhere to social norms (14, 15). These behavioral and neuropsychological changes often precede the development of region-specific brain atrophy on neuroimaging (6), leading to a low suspicion of bvFTD and delaying its diagnosis (16). Given these diagnostic challenges and the prominence of executive function and behavioral abnormalities in bvFTD, it is important to evaluate these neuropsychological markers by screening for executive dysfunction, social cognition disorders, and behavioral disturbances to distinguish bvFTD from psychiatric disorders (6, 17).

To improve the diagnostic accuracy of bvFTD, the use of brief psychological assessment tools evaluating social and emotional cognition has been proposed, particularly when cognitive screening tests that are routinely utilized in clinical practice appear to be normal or mildly abnormal (18–20). Tools such as the Social Cognition and Emotional Assessment (SEA) and its abbreviated version, the Mini-SEA, have demonstrated an ability to distinguish patients with bvFTD from controls (21–24) and bvFTD from major depressive disorders (22). The addition of other neuropsychological markers, such as social–emotional tasks and social–behavioral questionnaires, would improve the ability to distinguish between the early stages of bvFTD and early AD (6, 18, 21, 25), as these early alterations of the fronto-limbic circuitry are not observed in AD (26, 27).

Few research studies assessing bvFTD have been performed in Latin America, and a low prevalence of the disease throughout the region has been reported in one study (28). This low prevalence may be largely due to underreporting of and unfamiliarity with bvFTD in the primary care setting or among physicians lacking training in cognitive disorders (29, 30). Of the few studies that considered bvFTD, one study from Colombia found that behavioral disturbances were most common in patients with bvFTD but were also common in AD (31), emphasizing the need to tailor screening tests specific to the Latin American context to distinguish between these two entities. Various efforts have been made to combine cognitive and behavioral assessments for the detection of bvFTD with tests for social cognition and behavior, including global cognitive assessments (various versions of the Addenbrooke's Cognitive Examination, ACE), executive function (INECO Frontal Screening, IFS), and social cognition tests (32, 33). However, to date, there are no studies utilizing neurobehavioral scales that may help discriminate bvFTD from primary psychiatric disorders in Latin America.

Moreover, it is crucial to confirm this in low educational levels, as there are few reports of patients with bvFTD with low educational levels. One study from China found that educational levels were positively associated with a diagnosis of FTD and that patients with FTD tend to be more highly educated compared with patients with AD (34). For these reasons, bvFTD patients with lower educational levels are often not reported on. Therefore, we sought to compare the cognitive and socio-behavioral performance among Peruvian patients with a low educational level but who met the diagnostic criteria for bvFTD compared with a group who met the criteria for typical AD. We sought to evaluate the diagnostic accuracy of various neuropsychological markers that may be used to differentiate between the two neurodegenerative diseases, particularly in settings with a high prevalence of patients with low educational levels, such as in Peru.

## Methods

### Participants

A prospective study was performed, including 51 individuals, selected using convenience sampling, who presented for routine and regular neurological care at the Cognitive Impairment Diagnosis and Dementia Prevention Unit of the Instituto Peruano de Neurociencias (IPN) in Lima, Peru, between July 2017 and December 2020 (Supplementary Figure 1). These patients are followed regularly by their neurologist at the IPN, and following the study evaluation detailed below, they were classified into one of two study groups. Two groups of patients with low educational levels were studied: 33 patients with a diagnosis of typical AD and 18 with probable mild bvFTD after a diagnostic consensus using the gold-standard diagnostic criteria detailed below. The inclusion criteria were male or female individuals over 50 years of age who met the diagnostic criteria for dementia as per the DSM-V (35). The diagnosis of bvFTD was made by (1) a current revised diagnostic criteria from Rascovsky et al. (4) and (2) a clinical follow-up visit at least 2 years after the baseline visit confirming the initial diagnosis. The comparison group consisted of patients with a diagnosis of typical AD according to the published criteria from McKhann et al. (36). All participants had low educational levels (as described below) and mild to moderate cognitive impairment based on complete neuropsychological testing.

The exclusion criteria included the following: individuals with an inability to perform cognitive testing due to hearing or visual impairment or another physical health condition that interfered with performance, individuals whose primary language was not Spanish; individuals with a prior diagnosis of depression, individuals who had a stroke leading to cognitive deficit, individuals who had active psychiatric disorders, individuals who had a history of addiction or substance abuse, and individuals with cognitive impairment that could be explained by another cause, such as hypothyroidism, vitamin B12 deficiency, liver disease, chronic kidney disease, neurological infections (HIV-associated infections and syphilis), severe head trauma, and subdural hematoma. We excluded patients with severe dementia with complete dependence on a caregiver for activities of daily living, impairing their ability to complete the brief cognitive, and behavioral assessments. We also excluded individuals who, in the seven nights prior to the clinical evaluation, were taking the following medications: opioid analgesics, decongestants, anti-spasmodics, anti-emetics, anti-cholinergics, anti-arrhythmics, anti-depressants, anti-psychotics, anti-anxiety, or anti-epileptics. If the patients were chronically taking any of the aforementioned medications, cessation of the medication 7 days prior to the cognitive evaluation was recommended if safe to do so.

In addition, the participants of low educational levels were selected based on the following screening questions: First, the subjects were asked, “How many years of school did you attend?” Those who reported more than 6 years of formal education were excluded. Those who reported never attending school or completing <1 year of formal schooling were asked, “Are you able to read and write?” Those who reported not being able to read and/or write were excluded. Thus, our cohort was comprised of patients who had between 3 and 6 years of formal education.

### Ethical Considerations

All participants and their caregivers signed an informed consent form in accordance with the ethical guidelines for research with human subjects. The study protocol was approved by the institutional research ethics committee of the Hospital Nacional Docente Madre Niño San Bartolomé, CIEI 13184-17.

### Clinical and Neuropsychological Evaluation

The individuals underwent the following successive evaluations divided into three phases (screening, diagnosis of dementia, and designation of dementia type). During the screening phase, the individuals underwent a comprehensive clinical assessment and brief cognitive tests, including the Mini Mental State Examination (MMSE) (37), Clock Drawing Test—Mano's Version (PDR-M) (38, 39), and Pfeffer Functional Activities Questionnaire (PFAQ) (40). The individuals who scored below the threshold score for a diagnosis of dementia according to our inclusion criteria underwent a second assessment in which a second MMSE and PDR-M were administered by a different evaluator. The cutoff score on the MMSE for suspected dementia was adjusted according to the number of years of education of the patient: a score of 27 for individuals with more than 7 years of education (although no participants with more than 7 years of education were included in this study), 23 for those with 4 to 7 years of education, 22 for those with 1 to 3 years of education, and 18 for those who were illiterate. The PDR-M assesses the individual's ability to arrange the numbers 1 through 12 on a drawn circle as they would appear on a clock and then assesses the direction and proportionality of the clock's hands as they attempt to draw the time 11:10. The maximum score is 10, and in Peruvian individuals a score lower than 7 indicates cognitive impairment (38). The PFAQ includes 11 questions about activities of daily living, with scores ranging from 0 to 3 according to disability severity in each activity. The maximum score is 33, and a score >6 indicates functional dysfunction (40).

The individuals who were confirmed to have a “cognitive impairment” during the second round of testing then underwent blood tests (hemoglobin levels, glucose, urea, creatinine, liver function tests—AST and ALT, serum albumin, and globulin levels), vitamin B12 and folic acid levels, VDRL (to rule out syphilis), HIV ELISA, thyroid profile (T3, T4, and TSH), and serum electrolyte levels (sodium, potassium, and chlorine). These participants also underwent a brain MRI and depression screening using the Beck Depression Inventory-II to rule out pseudo-dementia and the Clinical Dementia Rating Scale (CDR). The sum of boxes on the CDR was applied to stage disease severity (41). In this second phase, a complete cognitive battery was administered by neuropsychologists (JC and CG) blinded to the clinical diagnosis of the patients. In the IPN, all patients were routinely administered this complete neuropsychological battery once yearly; thus, the results from the baseline and 2-year follow-up visit were used to determine the final dementia diagnosis. The battery consisted of the following tests: Rey Auditory Verbal Learning Test, Logical Memory Subtest of the revised Weschler Memory Scale, Trail Making Tests A and B, Rey Complex Figure, Boston Naming Test, Wisconsin Card Sorting Test, Letter-Number (subtest of the Weschler Adult Intelligent Scale III), Digit Span, Strub-Black Picture Copying, and the WAIS-III Cubes Test, as has previously been described (42). This battery also included an executive and social cognition battery consisting of five tests: Hotel Task, Multiple Errands Test—hospital version, Iowa gambling task, The Mind in the Eyes Test, and the Faux Pas Test.

In the last phase, the dementia type (AD or bvFTD) was determined by utilizing results from blood tests, neuroimaging, and complete neuropsychological testing by a consensus between neurologists (NC and MP-C), neuropsychologists (JC and CG), a neurorehabilitation specialist (RM), and a team psychiatrist (LC).

### Brief Cognitive, Social Cognition, and Behavioral Assessment Tests

These patients selected from these screening phases then went on to have the brief cognitive (ACE-III), social cognition (Mini-SEA), and behavioral assessments (FBI, IRI, and r-SMS) described below. The battery included measurements of global cognition (ACE-version III), executive function (IFS), social cognition (Mini-SEA), and behavioral symptoms (FBI: Frontal Behavioral Inventory; IRI-EC: Interpersonal Reactivity Index—Emphatic Concern; IRI-PT: Interpersonal Reactivity Index—Perspective Taking; r-SMS: Self-Monitoring Scale—revised version). The Mind in the Eyes Test and the Faux Pas Test were briefer versions of the original complete versions administered previously as part of the complete neuropsychological battery in the second screening phase. The brief social cognition and behavioral assessments tests were performed by evaluators different from those who administered the complete neuropsychological battery (VR-F—a medical epidemiologist and LM— a neuropsychologist) who were blinded to the results of the complete neuropsychological assessment. All scores used for analysis were from the baseline study visit.

#### Addenbrooke's Cognitive Examination, Version III

The Spanish version of the Addenbrooke's Cognitive Examination version III (ACE-III), adapted by a committee of expert investigators from Chile and Argentina, was used for this study (43). The test is comprised of five subscales (attention, memory, language, verbal fluency, and visuospatial skills) with a maximum score of 100. For each of the subscales, the following changes were made: the orientation and attention subscales were unified into one scale, and within them the question asking for the spelling of the word “WORLD” backwards was eliminated, leaving only the subtraction series of numbers. For the language subscale, the sentence “close your eyes” was removed, and the sentence writing task was changed to writing two sentences on a common theme. The complex commands were replaced by a three-step command with an increase in syntactic complexity; the two sentences previously used for the repetition test were modified; and in the naming test, the first two objects “watch and pencil” were replaced by two other familiar objects (spoon and book). In the visuospatial skills domain, the pentagons were replaced by intersecting infinity loops. The memory and verbal fluency domains were not modified.

#### INECO Frontal Screening

We used the Spanish version of the IFS validated for a Peruvian population (44). The IFS provides a detailed assessment of various executive functions (eight subtests), for a maximum of 30 points total (motor programming = 3, conflicting instructions = 3, motor inhibitory control = 3; reverse-order digit span = 6, verbal working memory = 2, spatial working memory = 4, abstraction = 3, and verbal inhibitory control = 6) where lower scores indicate a worse cognitive performance. The IFS begins by evaluating the motor series, asking the individual to consecutively perform the Luria series (fist, edge, and palm). Next, conflicting instructions and inhibitory motor control are evaluated by performing a series of instructions. Then, backwards digit repetition is evaluated, and verbal working memory is assessed by naming the months of the year backwards starting with the last month. For visual or spatial working memory, the individual is asked to point out the series of cubes drawn in reverse order of the one drawn by the evaluator. To evaluate abstraction, the individual is asked to interpret the meaning of three phases. Finally, to test for verbal inhibitory control, the individual is asked to complete an incomplete sentence with one word as quickly as possible (the initiation phase), while in the second phase (the inhibition phase), the individual is asked to complete the sentence with a word that does not make any sense in the context of the sentence.

#### Mini-Social Cognition and Emotional Assessment

We used the Spanish version of the Mini-social Cognition and Emotional Assessment (Mini-SEA) (22) adapted by Henriquez and collaborators for the Manual of Best Practices for the Diagnosis of Dementia (45). It is comprised of two subtests, the *faux pas* and the facial emotion recognition test. The *faux pas* assesses the theory of mind and consists of different “social” scenes that test the ability of a patient to detect social *faux pas* as well as explain why and how a *faux pas* occurred in each scene. Ten social scenes (plus one example scene) are presented in this subtest. The patient reads each story by himself/herself before the clinician asks a few questions about the story. The patient can read the story aloud, if preferred, and can re-read it at any time, including after each question. The facial emotion recognition test requires the patient to identify emotions from various faces. The patient is shown 35 male and female Caucasian faces and can choose from seven emotions for each face: happiness, surprise, neutral, sadness, fear, disgust, and anger.

#### Frontal Behavioral Inventory

The FBI is an informant-based behavioral questionnaire developed to identify bvFTD (46) and comprised of two subscales, one for negative symptoms (e.g., apathy, indifference, or loss of insight) and another for positive symptoms (inappropriate social behavior, aggression, or hyper-orality), with scores ranging from 0 to 72, where high scores indicate severe behavioral disturbances. The Spanish version of the FBI was used in this study (47).

#### The Interpersonal Reactivity Index

The IRI is comprised of four independent measures of seven items each: (a) “fantasy” which denotes a tendency of the subjects to identify with fictional characters such as book and movie characters (e.g., “after watching a play or movie, I feel as if I were one of the main characters”), (b) “perspective taking” which contains items that reflect the tendency or ability of the subjects to adopt the perspective or point of view of other people (e.g., “Sometimes I try to understand my friends better by imagining how they see things from their perspective”), (c) “empathic concern” which contains items that assessed the tendency of the subjects to experience feelings of compassion and concern toward others (e.g., “I often have feelings of compassion and concern toward people less fortunate than myself”), and (d) “personal distress” which includes items that indicated that the subjects experienced feelings of discomfort and anxiety when witnessing the negative experiences of others (e.g., “I sometimes feel helpless when I am in the middle of a very emotional situation”). Caregivers were interviewed to answer each of the 28 items that reflect on the behavior of the patient on a scale from 1 (does not describe the behavior of the patient) to 5 (describes the behavior of the patient very well) (48). The Spanish version of the IRI was used for this study (48).

#### Self-Monitoring Scale—Revised Version

The r-SMS is a questionnaire designed to assess the degree to which the subjects attend to the social-emotional cues of other individuals and allow these cues to influence their own behavior. This assesses the ability of the patients to adapt their behavior to a particular social context. It consists of subscales designed to measure the cognitive elements of empathy: the expressive behavior subscale which measures the sensitivity of the subjects to express the behavior of others and the self-presentation subscale which measures the tendency of the subjects to monitor their self-presentation. An informant (close relative) is asked to rate how well each of the 13 statements in the questionnaire describes the ability of the patient to modulate his or her behavior in various social situations on a six-point Likert scale (1 = certainly—always false to 6 = certainly—always true) (49). The validated Spanish version of the r-SMS was used (50).

### Statistical Analyses

We compared the results between patients with AD and those with bvFTD. We used descriptive statistics (means with standard deviations and proportions with absolute frequencies) to summarize numerical and categorical variables, respectively. We used Student's *t*-test and chi-square test, as appropriate, to assess the significance of differences between groups. We performed receiver operating characteristic (ROC) analysis to calculate the area under the curve (AUC) using the diagnosis of the patient as the gold standard to compare the brief screening tests of interest (ACE-III, Mini-SEA, IFS, FBI, IRI-EC, IRI-PT, and r-SMS) individually. In addition, we compared various combinations of these tests. We calculated the sensitivity, specificity, and percentage of correctly classified diagnoses for each cutoff point of the individual tests being compared. The analyses were performed with the statistical package STATA, version 16, with a significance level of 5%.

## Results

### Baseline Demographic and Cognitive Characteristics of the Study Groups

Fifty-one patients that met the inclusion criteria were included in this study. The AD group was significantly older than the bvFTD group (*p* < 0.001), but years of education (*p* = 0.4101), female sex (*p* = 0.394), and disease duration (*p* = 0.2407) were similar between groups. We also observed greater disease severity in patients with bvFTD measured by the CDR sum of boxes scale; however, the difference in disease severity between the two groups was not statistically significant (*P* = 0.8461; Table 1).

**Table 1:** Baseline demographic and clinical data of patients with Alzheimer's disease or behavioral variant of frontotemporal dementia (Instituto Peruano de Neurociencias, Lima; 2017–2020).

	**AD (*n* = 33)**	**bvFTD (*n* = 18)**	***P*-value (AD vs. bvFTD)**
Female, (%)	21 (63.64)	10 (55.56)	0.394
Age in years, mean (SD)	72.21 (3.48)	64.28 (5.44)	<0.001
Education in years, mean (SD)	4.79 (0.99)	4.72 (0.96)	0.4101
Disease duration in months, mean (SD)	38.21 (8.77)	36.56 (7.92)	0.2407
CDR sum of boxes score, mean (SD)	3.88 (1.65)	4.39 (1.76)	0.8461
ACE-III score, mean (SD)	70.33 (4.53)	62.61 (5.87)	<0.001
IFS score, mean (SD)	20.12 (2.09)	17.17 (3.43)	0.0014^*^
Mini-SEA score, mean (SD)	21.55 (1.33)	16.06 (2.51)	<0.001
FBI score, mean (SD)	9.88 (3.71)	24.83 (5.09)	<0.001
IRI-EC score, mean (SD)	24.56 (2.53)	20.5 (2.28)	<0.001
IRI-PT score, mean (SD)	18.33 (2.39)	13.11 (1.91)	<0.001
r-SMS score, mean (SD)	39.42 (3.60)	30.72 (3.71)	<0.001

*AD, Alzheimer's disease; bvFTD, behavioral variant of frontotemporal dementia; SD, standard deviation; CDR, Clinical Dementia Rating scale; FTD, frontotemporal; ACE-III, Addenbrooke's Cognitive Examination—version III; IFS, INECO Frontal Screening; Mini-SEA, Mini-social Cognition and Emotional Assessment; FBI, Frontal Behavioral Inventory; IRI, Interpersonal Reactivity Index; IRI-EC, Empathic Concern Subscale of IRI questionnaire; IRI-PT, Perspective Taking subscale of the IRI questionnaire; r-SMS, revised-Self Monitoring Scale*.

* *p-value < 0.05*.

**Figure 1:**
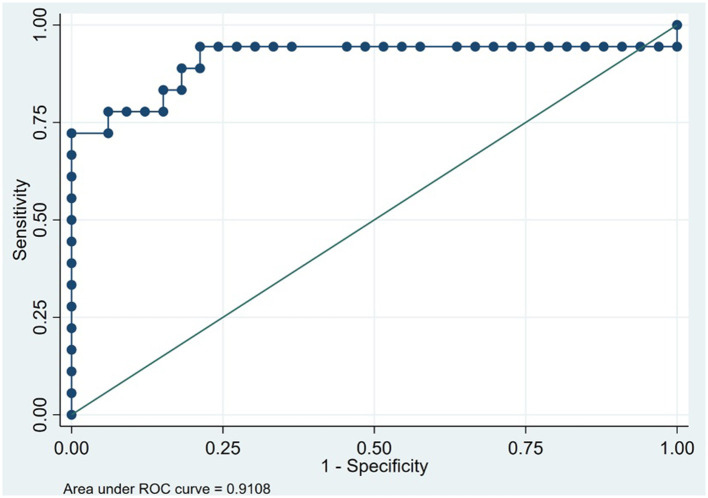
Receiver operating characteristic curve for the Addenbrooke's Cognitive Examination plus the INECO frontal screening in 51 patients to discriminate between behavioral variant frontotemporal dementia and Alzheimer's disease (Instituto Peruano de Neurociencias, Lima, Peru; 2017–2020).

All scores reported are from the baseline visit. The bvFTD group performed significantly worse in global cognitive assessment scores compared with the AD group in both the ACE-III total score (*p* < 0.001) and the IFS (*p* < 0.001; Table 1). However, an analysis of the discriminatory ability of the ACE-III to distinguish between patients with AD and those with bvFTD (area under the ROC curve = 0.85) and the IFS (area under the ROC curve = 0.78) shows that the former has greater discriminatory ability to distinguish patients with bvFTD from those with AD (Table 2). For the ACE-III total score, a cutoff score of 70 had a sensitivity of 67% and a specificity of 94%. For the IFS, a cutoff score of 19 demonstrated a sensitivity of 76% and a specificity of 67%, but when the ACE-III was combined with the IFS, there was a slight increase in its discriminatory capacity (Figure 1; Table 2).

**Table 2:** Baseline cutoff scores and diagnostic performance for the global cognition, social cognition, and behavioral tests to discriminate patients with Alzheimer's disease and behavioral variant frontotemporal dementia (Instituto Peruano de Neurociencias, Lima, Peru; 2017–2020).

	**Cutoff score**	**AUC**	**Sensitivity, %**	**Specificity, %**	**Correctly classified, %**	**LR+**	**LR–**
ACE-III	70	0.85	66.67	94.44	76.47	12.00	0.3529
IFS	19	0.78	75.76	66.67	72.55	2.27	0.3636
Mini-SEA	19	0.96	100	83.33	94.12	6.00	<0.001
ACE-III + IFS		0.91	77.78	90.91	86.27		
ACE-III + IFS + Mini-SEA		0.96	88.89	100	96.08		
FBI	19	0.5	83.33	100	94.12		0.1667
IRI-EC	22	0.89	87.88	66.67	80.39	2.64	0.1818
IRI-PT	16	0.97	93.94	88.89	92.16	8.45	0.0682
r-SMS	32	0.95	100	72.22	90.20	3.60	<0.001

*AUC, area under curve; LR+, positive likelihood ratio; LR-, negative likelihood ratio; ACE-III, Addenbrooke's Cognitive Examination-version III; IFS, INECO Frontal Screening; Mini-SEA, Mini Social Cognition and Emotional Assessment; FBI, Frontal Behavioral Inventory; IRI, Interpersonal Reactivity Index; IRI-EC, Empathic Concern subscale of IRI questionnaire; IRI-PT, Perspective Taking subscale of IRI questionnaire; r-SMS, revised-Self Monitoring Scale*.

### Other Neuropsychological Markers

**Figure 2:**
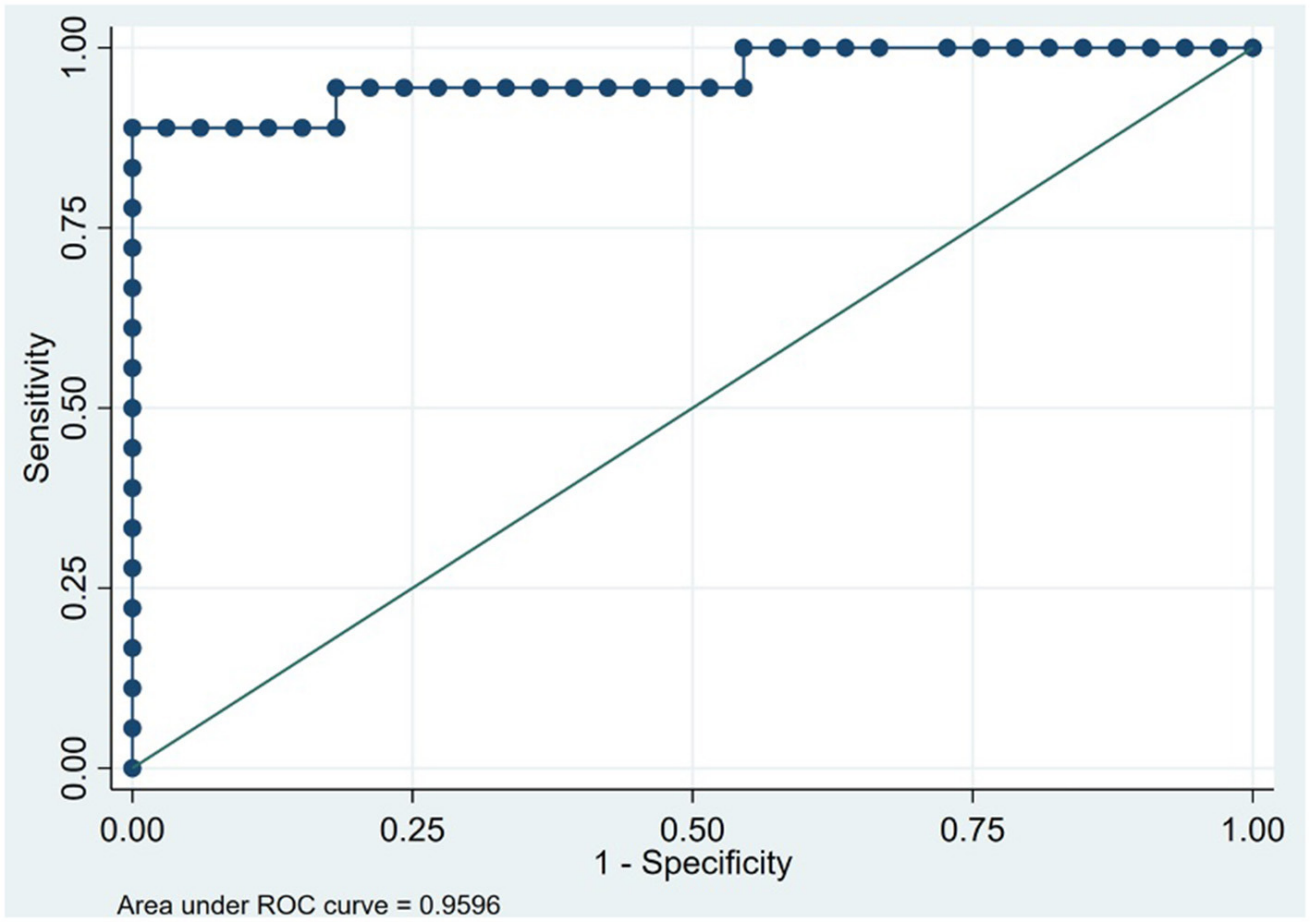
Receiver operating characteristic curve for the Addenbrooke's Cognitive Examination plus the INECO frontal screening plus Mini-social Cognition and Emotional Assessment in 51 patients to discriminate between behavioral variant frontotemporal dementia and Alzheimer's disease (Instituto Peruano de Neurociencias, Lima, Peru; 2017–2020).

**Figure 3:**
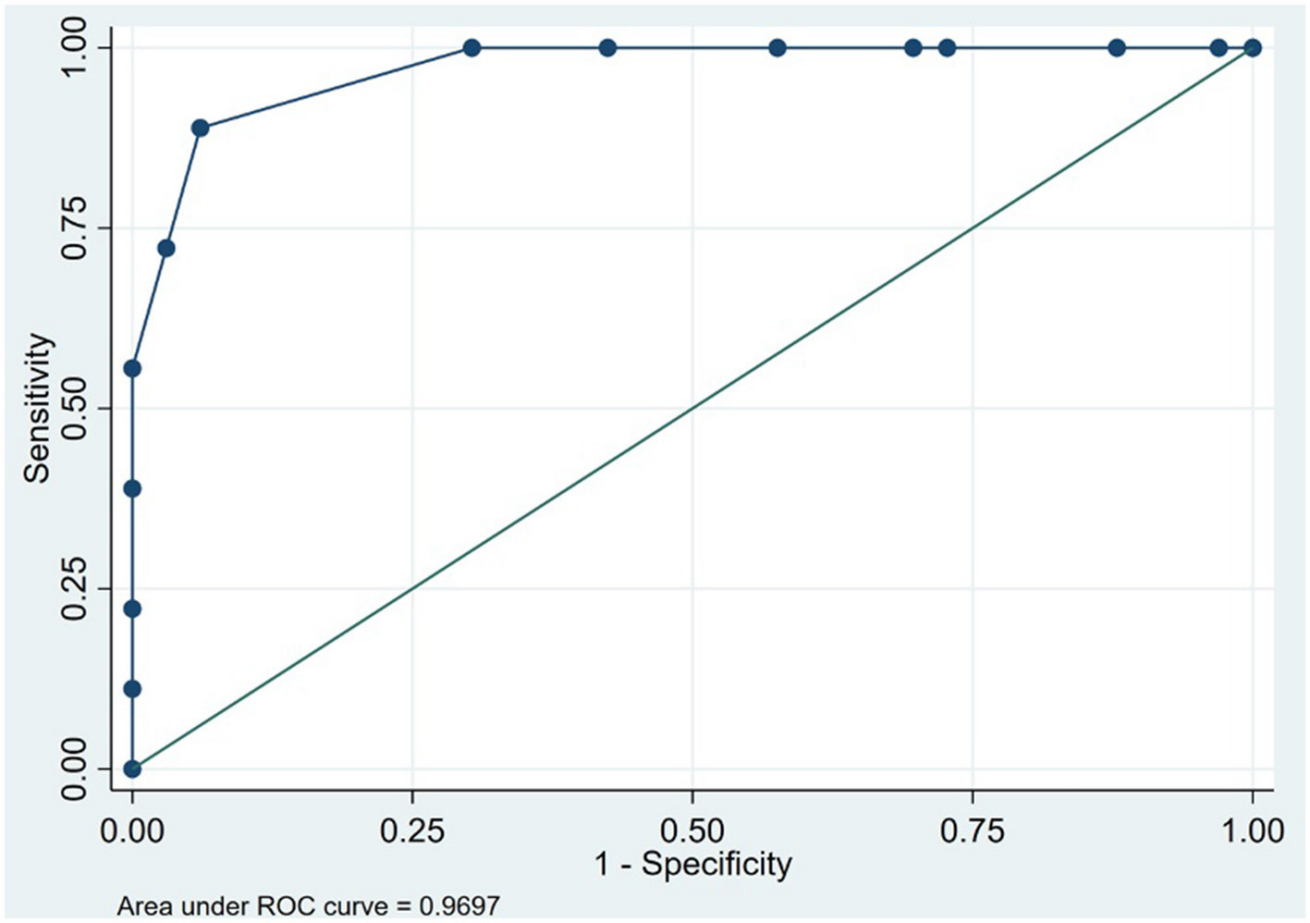
Receiver operating characteristic curve for Interpersonal Reactivity Index, Perspective Taking subscale in 51 patients to discriminate between behavioral variant frontotemporal dementia and Alzheimer's disease (Instituto Peruano de Neurociencias, Lima, Peru; 2017–2020).

**Figure 4:**
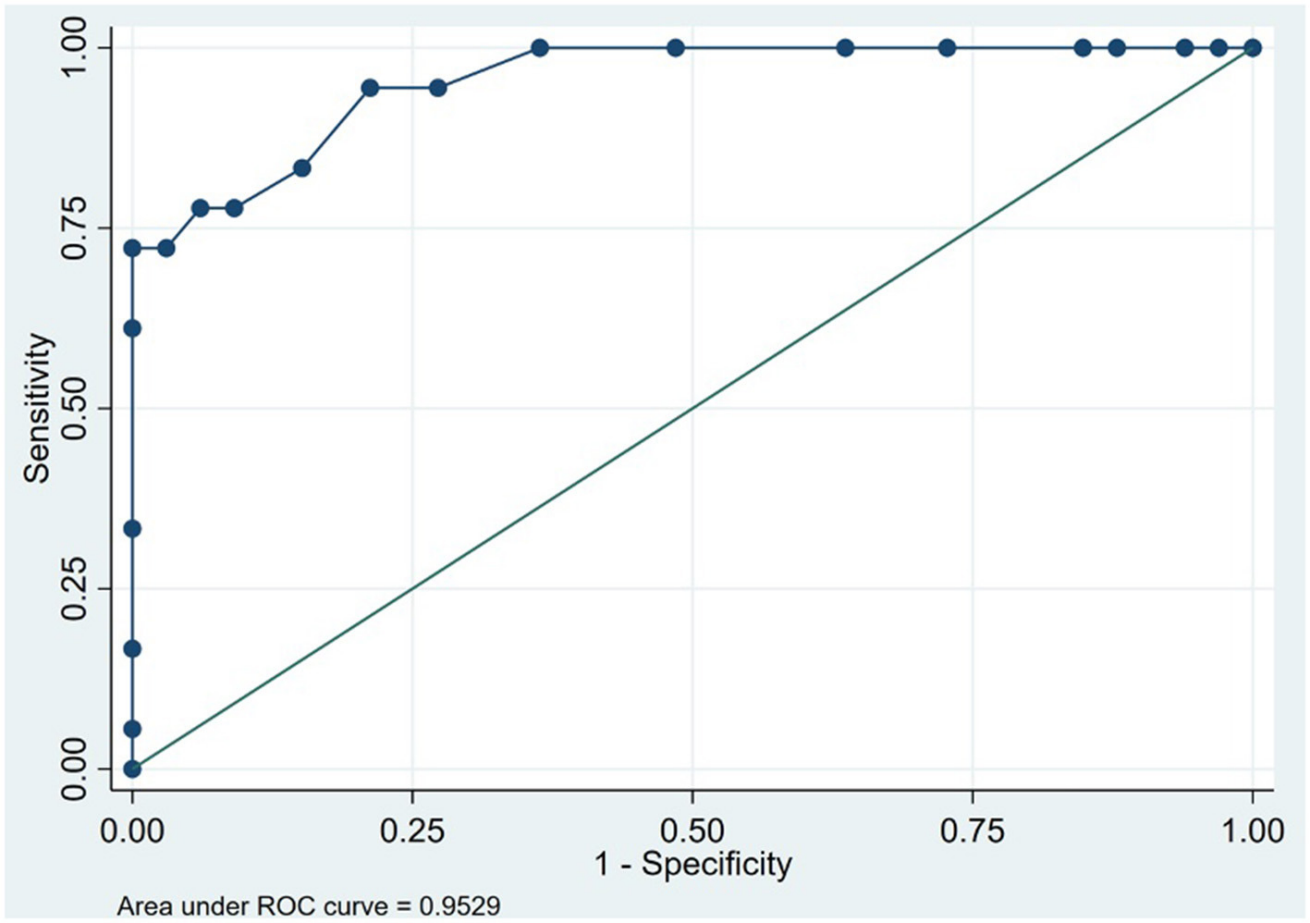
Receiver operating characteristic curve for revised-Self Monitoring Scale in 51 patients to discriminate between behavioral variant frontotemporal dementia and Alzheimer's disease (Instituto Peruano de Neurociencias, Lima, Peru; 2017–2020).

The social and behavioral cognition tasks were able to appropriately discriminate bvFTD from AD. The bvFTD group performed significantly worse (*p* < 0.001) on the Mini-SEA compared with the AD group (Table 1). The sensitivity of the Mini-SEA for discriminating between bvFTD and AD was ideal with high moderate specificity (83%), which increased when combined with the brief screening tests ACE-III and IFS (Figure 2, Table 2). As expected, behavioral changes characterized patients with bvFTD to a greater degree than those with AD. The bvFTD group performed significantly worse on the FBI (higher mean scores) compared with the AD group (*p* < 0.001; Table 1). The specificity of the FBI was ideal with high sensitivity (83%) and reflected the severe social cognition impairment of the patient as judged by their caregivers (Table 2). The bvFTD group had significantly lower scores, representing worse performance, in both the IRI-EC and IRI-PT (*p* < 0.001 for both tests; Table 1). The sensitivity of the IRI-EC was high with moderate specificity and with ideal AUC, and the IRI-PT also demonstrated high sensitivity and specificity for distinguishing bvFTD from AD (Table 2, Figure 3). The r-SMS had an ideal AUC and sensitivity with moderate specificity (Figure 4), demonstrating its less ability to adapt behaviorally to a given social situation.

## Discussion

In our study, we evaluated the combined utility of social cognition and social–behavioral tools in diagnosing bvFTD among a sample of patients living in an urban setting with low educational levels from a developing country. Although both groups had statistically similar disease severity (based on the CDR sum of boxes), the bvFTD group performed worse on global cognitive assessment (ACE-III) and executive function assessment (IFS) compared with the AD group. As expected, the bvFTD group had more significant impairment in behavioral scales (FBI, IRI-EC, and IRI-PT), all with high sensitivity in differentiating bvFTD from AD. We also found that the sensitivity for detection of bvFTD was greatest when combining the Mini-SEA, ACE-III, and IFS in a population of Peruvian patients with <6 years of formal education and would also be less time-consumptive (about 50 min) to administer compared with a complete neuropsychological battery.

The recent guidelines for the diagnosis of bvFTD include the administration of at least one social cognition or social–behavioral task embedded within a standard neuropsychological battery. Other recommendations include using validated visual atrophy rating scales and volumetric analyses of brain regions on MRI, 18F-fluorodeoxyglucose PET, neurofilament light chain in serum or cerebrospinal fluid (CSF), and screening for C9orf72 mutation in patients with prevalent psychiatric symptoms (6). However, these guidelines are not standards of care in most clinical settings worldwide and are reserved for research purposes or for cases in which the clinical diagnosis is unclear based on clinical presentation or neuropsychological testing (51). Despite the importance of these diagnostic modalities, there are significant logistical challenges in their practical application for the diagnosis of bvFTD across Latin America and throughout many low- and middle-income countries (LMIC) that have limited access to these resources. Therefore, it is crucial to adapt and validate brief social cognition and behavioral tests that are easily applicable in the clinical setting and may decrease the frequency of false negatives in diagnosing bvFTD, particularly among those with lower educational levels.

In our study, the ACE-III, a test of global cognitive function, demonstrated better specificity than the IFS in discriminating patients with bvFTD from AD in low educational levels. However, in our study, the cutoff score with the best specificity was 70, well-below a score of 88 previously found to discriminate patients with degenerative dementias (including FTD) from those with depressive disorders (52) (to date, there are no studies in the literature that have identified the ideal cutoff score for distinguishing between AD and bvFTD). This lower ideal cutoff may be explained by the mean low educational level of our population, with most participants not having completed up to only primary school. Similar effects on the ideal cutoff of the ACE-III for differentiating between AD and cognitively healthy controls have been previously described among a sample of patients from Argentina with low educational levels (53). Alternatively, the IFS, an executive function-specific cognitive screening tool, provides valuable information on the early deterioration of executive function abilities in degenerative and psychiatric conditions (54). Patients with bvFTD perform worse in several sub-items of the IFS compared with patients with major depressive disorder and bipolar disorder (54). Additionally, educational levels are known to influence the IFS results (55, 56), making it an ideal tool in LMIC given the high prevalence of individuals with low educational levels. The Mini-SEA developed by Bertoux et al. has adequate sensitivity in the detection of ventromedial prefrontal dysfunction in patients with bvFTD (23) compared with the classical executive function tasks (57). These changes usually precede the onset of the dysexecutive syndrome that develops later in bvFTD (difficulty with planning, abstract thinking, and behavioral control) (58). Our study suggests that patients with bvFTD consistently perform poorly on these social cognition tools, supporting the ability of these tools to distinguish bvFTD from AD. Although the specificity of the Mini-SEA increased when combined with the ACE-III and the IFS, given the administration time of all three combined tests and the time constraints of physicians in developing countries, the most practical approach may be a combination of the IFS and the Mini-SEA as the first screening tools for the detection of bvFTD.

Social cognition includes several domains affected in bvFTD, including emotion recognition (cognitive and affective), theory of mind, empathy, and moral judgment (59). Theory of mind tasks, such as Reading the Mind in the Eyes test (included within the Mini-SEA) (57), are useful for the detection of FTD, particularly for longitudinal assessments of FTD (60), and related neurodegenerative disorders, such as amyotrophic lateral sclerosis (ALS) (61). Importantly, our findings support the utility of the Mini-SEA tasks in the neuropsychological evaluation of patients with suspected bvFTD for a more precise and early diagnosis. However, social cognition tasks can be altered in other FTD disorders, such as in the semantic variant of primary progressive aphasia or cortico-basal degeneration (62, 63), in AD (64), and in bipolar disorders, posing a problem to their application (65). Therefore, the sensitivity of social cognition screening tools, such as the FBI, IRI, and r-SMS, is important to explore in various populations, including those with low educational levels, as they may serve as an early diagnostic screening tool for bvFTD in assessing socio-behavioral changes by evaluating responses to real-life situations (20, 59, 66).

As expected, patients with bvFTD more often presented with severe behavioral disturbances, with at least 50% of our study group with bvFTD meeting the cutoff point for frontal behavioral syndrome on the FBI. Although the FBI is considered an efficient and accurate scale for early diagnosis of bvFTD (67), the proposed cutoff point of 19 was ineffective for the detection of bvFTD in our study (AUC 0.5 in our study); a finding similar to that was found in a study in Italy (46), in which a cutoff point of 23 was suggested for bvFTD detection (6, 67, 68). Moreover, the total score on the FBI does not distinguish between bvFTD and primary psychiatric disorders; however, specific FBI sub-items that support this distinction include aphasia and verbal apraxia, emotional indifference/flattery, foreign hand, and inappropriate social behavior (inappropriateness), whereas irritability has been found to be indicative of a primary psychiatric disorder (68). In our sample of patients with bvFTD, the IRI was able to measure empathy deficit, consistent with the findings previously published by Eslinger et al. (26). Analyzing regional brain atrophy patterns (62), functional connectivity (69) and pathological studies may demonstrate the relationship between loss of emotional empathy (measured by the IRI-EC) and alteration of specific neuronal networks among patients with bvFTD and ALS (70). In our study, the IRI-PT subscale achieved better discriminative ability than the IRI-EC, which is likely because patients with bvFTD have impaired self-monitoring skills (66, 71, 72). Our study demonstrated that the r-SMS has good discriminative ability to detect socio-emotional symptoms even at mild stages and proves to be optimal for screening as early r-SMS changes are sensitive to disease progression (73).

Cognitive dysfunction and socio-behavioral changes typical of bvFTD reflect the extent of neuronal damage and regional cerebral atrophy of the ventro-medial and dorso-lateral pre-frontal cortex, areas responsible for socio-behavioral conduct (74). This is also seen in brain networks responsible for social cognition, including a network involving the anterior insula, anterior cingulate, lateral orbitofrontal, amygdala, thalamus, and peri-aqueductal gray (66, 70) and the semantic appraisal (limbic) system (75, 76). The results of our analysis suggest that a combination of cognitive (global cognition and social cognition total scores) and behavioral (frontal and social–emotional behavioral change total scores) measures is the best neuropsychological marker for screening for bvFTD and may be used as an adjunct to the clinical and standard neuropsychological batteries for the diagnosis of bvFTD. Importantly, the detection of social–behavioral changes are crucial for the early and timely identification of bvFTD, given the high sensitivity of these symptoms in the diagnosis of bvFTD; however, they may be insufficient to differentiate this syndrome from other neurodegenerative conditions because of their low to moderate specificity (17, 25). Individually, none of the global cognitive tests alone are sufficient to provide data for the diagnosis of bvFTD (17, 19). However, the ACE-III seems to have high discriminatory capacity to distinguish between bvFTD and AD (52, 77), but given its long administration time and the use of pencil and paper, it poses challenges to implementation within the primary care setting (78), particularly among low-education and low-literacy populations. Therefore, briefer tests in combination that increase the sensitivity for the detection of bvFTD are needed. Our findings suggest that a test for executive function (IFS) combined with a social cognition test (Mini-SEA) and a social-emotional test (r-SMS) could improve the diagnostic and discriminative capacity of patients with cognitive impairment in situations where memory is not the predominant feature at symptom onset, as is often seen in bvFTD.

Our study has some limitations that are worth noting. First, the gold standard for the diagnosis of AD and bvFTD was based on clinical history, MRI brain imaging, and complete neuropsychological testing, without access to pathological, genetic, or CSF studies (recommended for the diagnosis of bvFTD) (6), which could limit the implications of our findings. However, we utilized the most sensitive brief cognitive and specialized neuropsychological tests that have been previously validated in our population at our clinic, MRI brain findings, and re-assessed the patients at 2-year follow-up to ensure that the diagnosis of bvFTD was accurate (44, 78, 79). These diagnostic criteria have been utilized in other studies of patients with bvFTD (80, 81). Additionally, no bvFTD cases had temporo-parietal damage associated with frontal atrophy on MRI, a typical pattern of frontal variant AD (82), further supporting the correct classification of patients. In addition, we ensured the appropriate diagnosis of bvFTD by including a clinical evaluation 2 years after the baseline visit, as the diagnosis is often made over time (6, 74). We also could not determine if there were age-related effects on the brief tests administered, which may be a limitation. A second limitation of the study worth noting is the lack of validation of the behavioral and socio-emotional assessment tools applied in this study within Peru and within our specific population of persons with low educational levels living in an urban environment. However, we applied the Spanish versions of these tools that have been previously validated in Latin American countries with a similar sociocultural context as that of Peru (47, 48, 50). Third, our small sample size is a limitation worth noting, limiting the generalizability of our results to populations different from that of our study. However, there is an overall low prevalence of FTD, and FTD is particularly difficult to diagnose in resource-limited settings without access to MRI due to financial constraints. Despite these challenges, to our knowledge, this is the first study in the literature to analyze socio-emotional and behavioral screening tools to distinguish AD from bvFTD in a population of persons with low education. Next, we excluded persons living in rural areas and persons with a native language other than Spanish; thus, our results cannot be extrapolated to these vulnerable populations. Lastly, this was not a prospective validation study, limiting the applicability of our results into clinical practice.

In conclusion, our study supports the integration of socio-behavioral measures to the standard global cognitive and social cognition measures utilized for screening for bvFTD in a population with low levels of education. This is particularly useful in primary care settings, given their easy applicability and shorter administration time. Our findings suggest that a combination of tests—the Mini-SEA, r-SMS, and IFS—could improve the diagnostic and discriminative capacity of patients with cognitive impairment and behavioral symptoms. This combination of tests may increase the detection of cases in the Latin American region where a “low prevalence” of bvFTD was previously suspected, largely due to underreporting or misclassification of the condition (28). However, a larger prospective validation study of these tools in our population is warranted for further confirmation of our findings. Using these screening tests may help reduce the need for neuroimaging (MRI or PET), particularly in LMIC with less access to these modalities, may help reduce healthcare costs, may increase the early identification of this condition, and may increase awareness in the medical community of bvFTD.

## Data Availability Statement

The raw data supporting the conclusions of this article will be made available by the authors, without undue reservation.

## Ethics Statement

The studies involving human participants were reviewed and approved by Hospital Nacional Docente Madre Niño San Bartolomé, CIEI 13184-17. The patients/participants provided their written informed consent to participate in this study.

## Author Contributions

NC, RM, and LC: scientific concept, drafting of manuscript, critical revision for scientific content. EH-P and VF-R: statistical analyses, drafting of manuscript, critical revision for scientific content. MP-C, WS, JC, and CG: data collection, drafting of manuscript, critical revision for scientific content. MD: drafting of manuscript, critical revision for scientific content. All authors contributed to the article and approved the submitted version.

## Conflict of Interest

The authors declare that the research was conducted in the absence of any commercial or financial relationships that could be construed as a potential conflict of interest.

## Publisher's Note

All claims expressed in this article are solely those of the authors and do not necessarily represent those of their affiliated organizations, or those of the publisher, the editors and the reviewers. Any product that may be evaluated in this article, or claim that may be made by its manufacturer, is not guaranteed or endorsed by the publisher.
