# Supplementary figures and images for: Social Cognition and Behavioral Assessments Improve the Diagnosis of Behavioral Variant of Frontotemporal Dementia in Older Peruvians With Low Educational Levels
Source: Front Neurol. 2021 Sep 6;12:704109. doi: 10.3389/fneur.2021.704109 (PMC8450322; doi:10.3389/fneur.2021.704109)

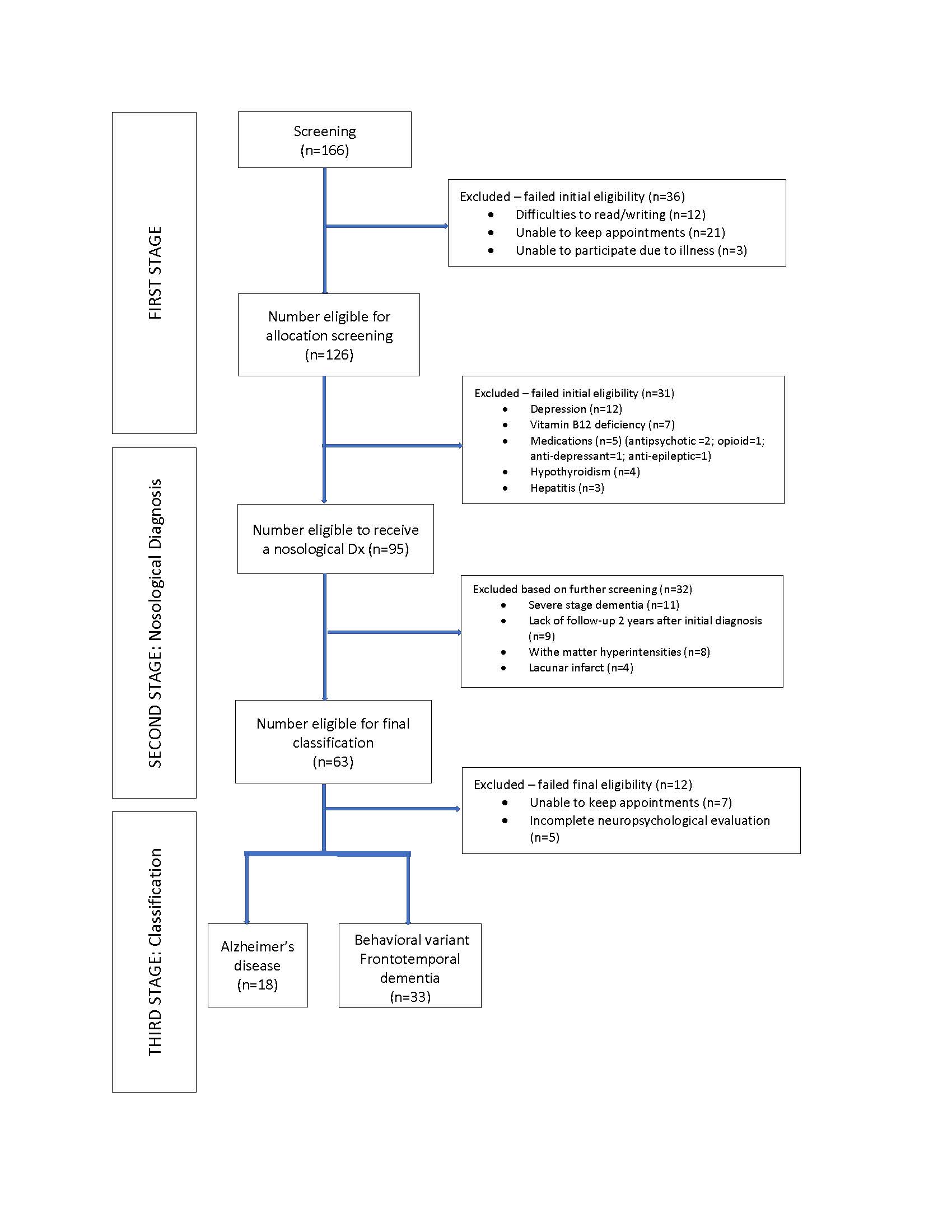

Supplement: Supplementary file 1 [file Image_1.jpg]
